# Streamlining robotic-assisted abdominoperineal resection

**DOI:** 10.1186/s12957-023-03260-x

**Published:** 2023-12-20

**Authors:** Kazunosuke Yamada, Jun Imaizumi, Ryuji Kato, Takahiro Takada, Hitoshi Ojima

**Affiliations:** Department of Gastroenterological Surgery, Gunma Prefectural Cancer Center, 617-1, Nishimachi, Oota-Shi, Gunma 373-0828 Japan

**Keywords:** Robotic surgery, Abdominoperineal resection, Resource management

## Abstract

**Background:**

Robot-assisted surgery has proven to be a safe and feasible approach for the management of rectal cancer, including abdominoperineal resection (APR). However, it often incurs longer operative times and higher costs. This study aimed to overcome these limitations by adopting a synchronous approach utilizing an optimized team composition.

**Methods:**

Data on patients who underwent robot-assisted APR at our facility between June 2022 and June 2023 were analyzed. The key points of the optimized approach included the following: At the start of the surgery, the surgeon performed an anococcygeal ligament resection from the perineal side while the bedside assistants set up the ports. Then, through console manipulation, the presacral fascia, elevated by previously placed gauze, was easily and safely incised, providing access to the perineal region.

**Results:**

A total of nine patients were included in this study. The median operation time was 231 min, and the intraoperative blood loss was 170 ml. The operation time was reduced to 167.5 min, and the blood loss was 80.5 ml in cases without a trainee. Surgical site infections, classified as Clavien–Dindo grade II complications, were observed in two cases, but no obvious urinary or erectile dysfunction was observed.

**Conclusion:**

The study results indicate that the challenges associated with APR can be efficiently addressed without requiring additional personnel by streamlining team composition and the synchronous approach. This optimization strategy minimizes the need for a larger surgical team, while maximizing the utilization of surgical time and resources.

## Introduction

Abdominoperineal resection (APR) is the preferred surgical method for advanced lower rectal cancer with large tumors, sphincter invasion, or challenges in achieving an adequate distal resection margin [1]. Compared with other rectal surgeries, APR involves more complex surgical techniques in the deeper pelvis and requires access to the deepest part of the pelvic cavity. These complexities increase the risk of a positive circumferential resection margin (CRM), which can result in local recurrence [2].

Robot-assisted surgery has proven to be a safe and feasible approach for the management of rectal cancer, including APR [2–6]. Several studies have demonstrated its technical viability and oncological effectiveness, especially in challenging cases with obesity or narrow pelvic anatomy [7–10]. However, robot-assisted surgery has some drawbacks, including longer operative times and higher costs. The adoption of robotic technology in surgery is rapidly increasing. However, there are concerns about the initial implementation costs and the ongoing high operational costs associated with this approach [9, 11, 12].

Therefore, a synchronous approach was developed to address the challenges associated with APR, including reducing surgical time and overcoming technical difficulties. This approach enables two surgical teams to simultaneously perform abdominal and perineal dissections [13, 14]. However, a limitation of this approach was the requirement for a larger surgical team consisting of four or more surgeons. In our facility, we have streamlined this surgical approach and mitigated this problem by having the surgeon perform part of the perineal manipulation while the assistants prepare for docking the patient cart. This paper aims to highlight the positive results achieved with this approach.

## Material and methods

### Patients and study design

This was a retrospective study. Data on 99 patients with rectal cancer who underwent robot-assisted APR at Gunma Prefectural Cancer Center between June 2022 and June 2023 were analyzed. In September 2019, the implementation of robot-assisted rectal resection began at our institution. From January 2020 onwards, robot-assisted surgery was adopted as the standard approach for all rectal cancer cases. By June 2022, the implementation of optimized robot-assisted APR was gradually established, enhancing the efficiency of the procedure. Cases involving lateral lymph node dissection were excluded from the analysis to evaluate the surgical time. The procedures were performed by two experienced colorectal surgeons, who independently operated on the cases. All aseptic maneuvers during the surgeries were performed by two bedside assistants in training. Additionally, another trainee participated as the console surgeon under the dual-console system, receiving guidance from experienced surgeons.

Clinical, pathological, and perioperative data were collected from our institution’s surgical database. CRM involvement was defined as the presence of tumor cells located 1 mm or less from the painted resection margin as determined microscopically. Complications were classified according to the Clavien–Dindo classification [15], and major complications were defined as grade II or higher. Postoperative complications were defined as the occurrence of adverse events within 30 days after surgery. Local recurrence-free survival (LRFS) was defined as the duration in which patients remained free from local recurrence post-surgery.

### Surgical technique

**Fig. 1:**
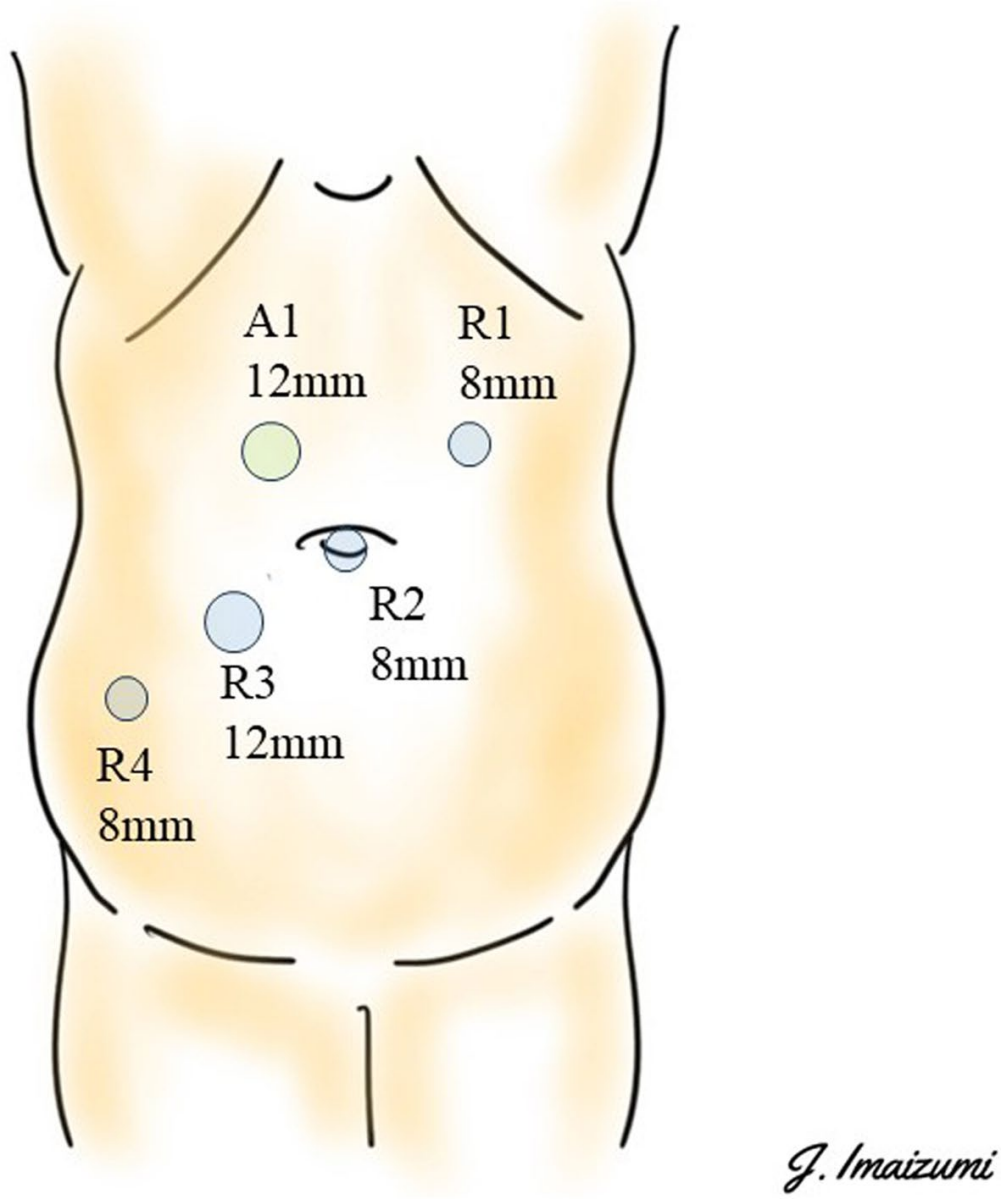
Trocar placement for robotic abdominoperineal resection

Patients were consistently placed in the Trendelenburg position from the beginning to the end of the surgery. In principle, three 8-mm robotic ports, one 12-mm robotic port, and one laparoscopic trocar were placed (Fig. 1).

All pelvic operations were performed using a dual-console four-arm robotic system (Da Vinci® Xi, Intuitive Surgical®), with fenestrated bipolar forceps attached to arm 1, monopolar curved scissors attached to arm 3, and tip-up fenestrated grasper attached to arm 4.

### The first half of perineal manipulation (Fig. 2)

**Fig. 2:**
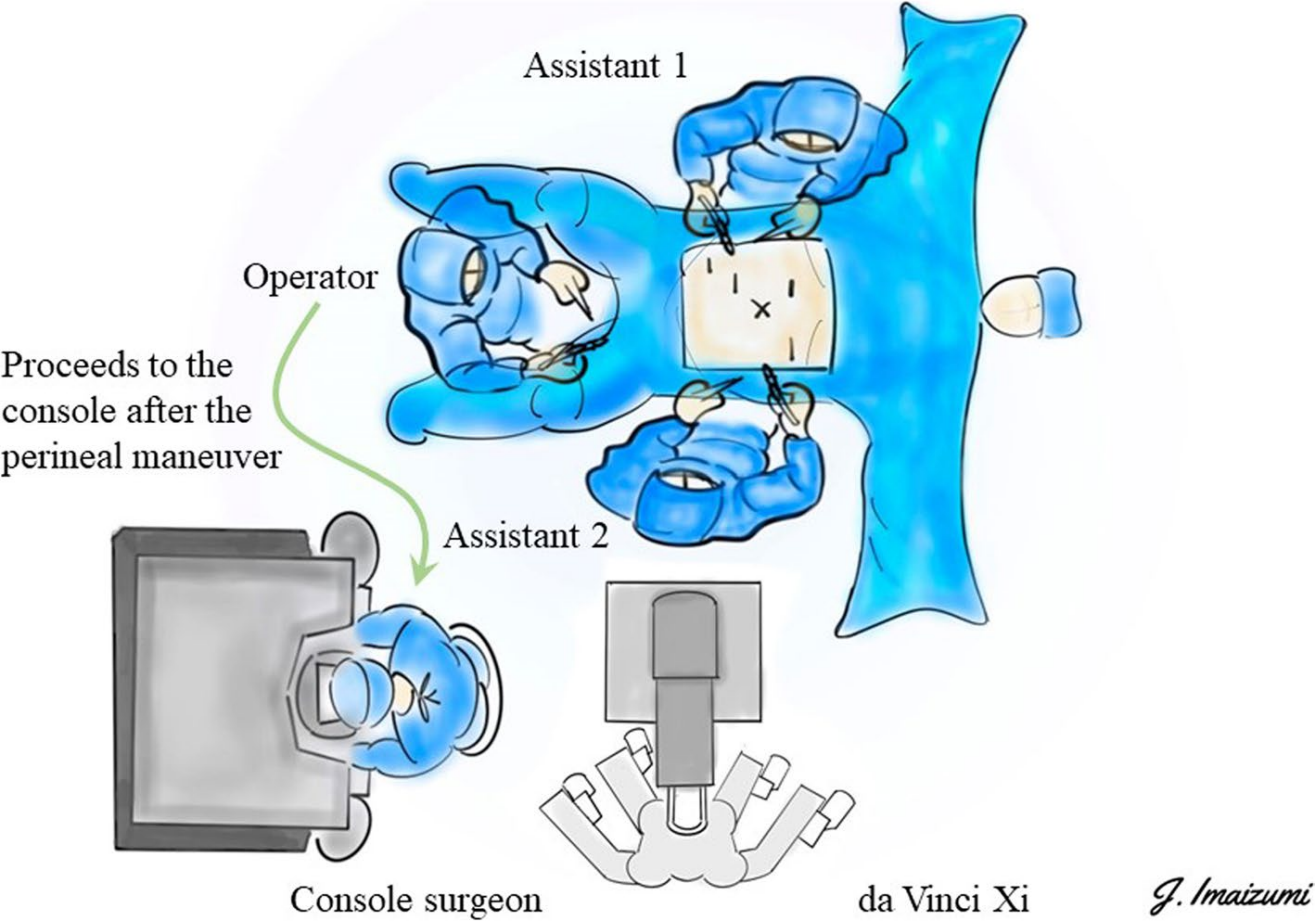
Surgical team positioning during operation

**Fig. 3:**
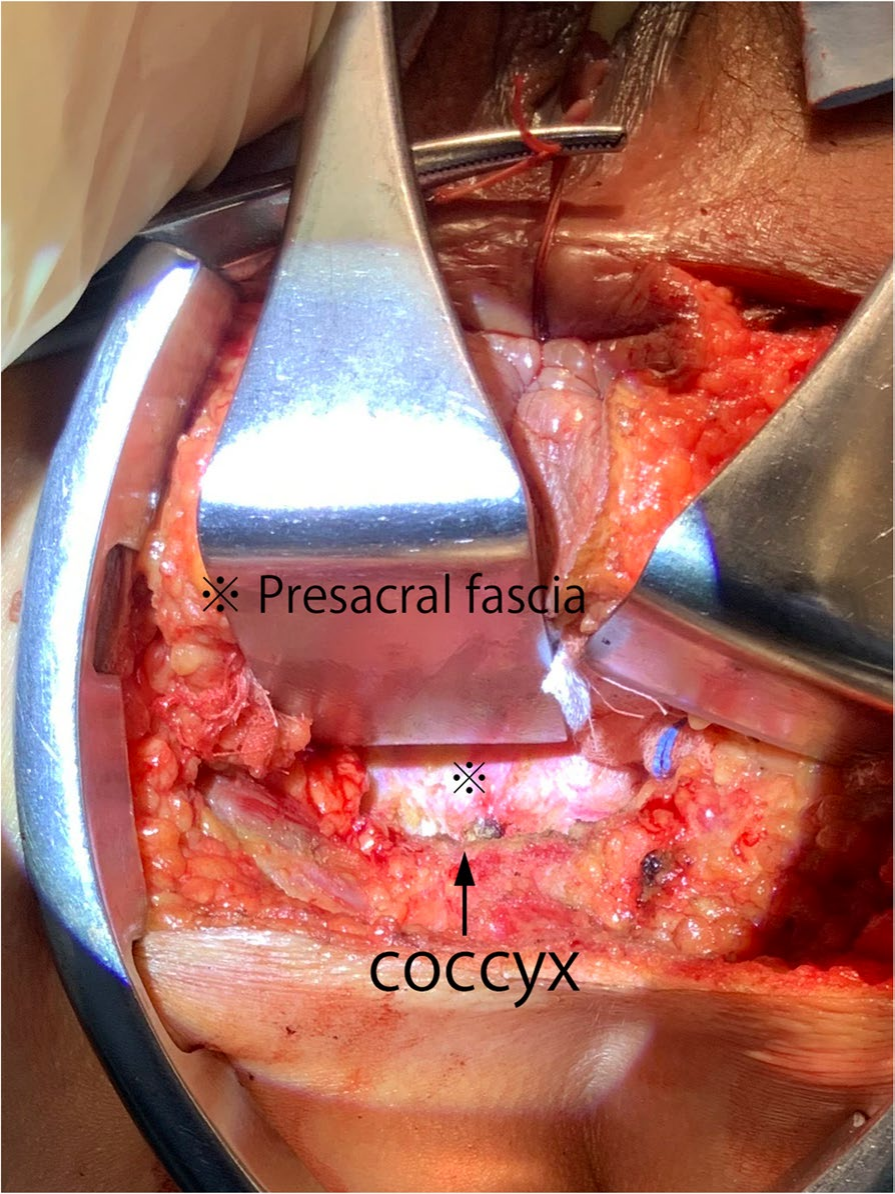
Resection of the anococcygeal ligament during the first half of the perineal manipulation

1. The anus was carefully sutured with a purse-string stitch to prevent fecal leakage. After thorough disinfection, the surgery began.
2. Simultaneously with the placement of ports by the bedside assistants, the surgeon performed a circular skin incision with a radius of approximately 3 cm around the anus.
3. An incision was made through the subcutaneous fat using the bilateral ischial tuberosities and coccyx as landmarks.
4. The incision on the anterior wall side, involving the superficial transverse muscle of the perineum and the puborectalis, was kept to a minimum. Emphasis was placed on the coccyx, with the incision extended toward the intrapelvic side.
5. Sharp resection of the anococcygeal ligament at the distal end of the coccyx enabled visualization of the presacral fascia (Fig. 3).
6. Gauze was placed in the plane, and the first half of the perineal operation was completed.

These steps were carried out until the docking procedure began.

### Console manipulation

**Fig. 4:**
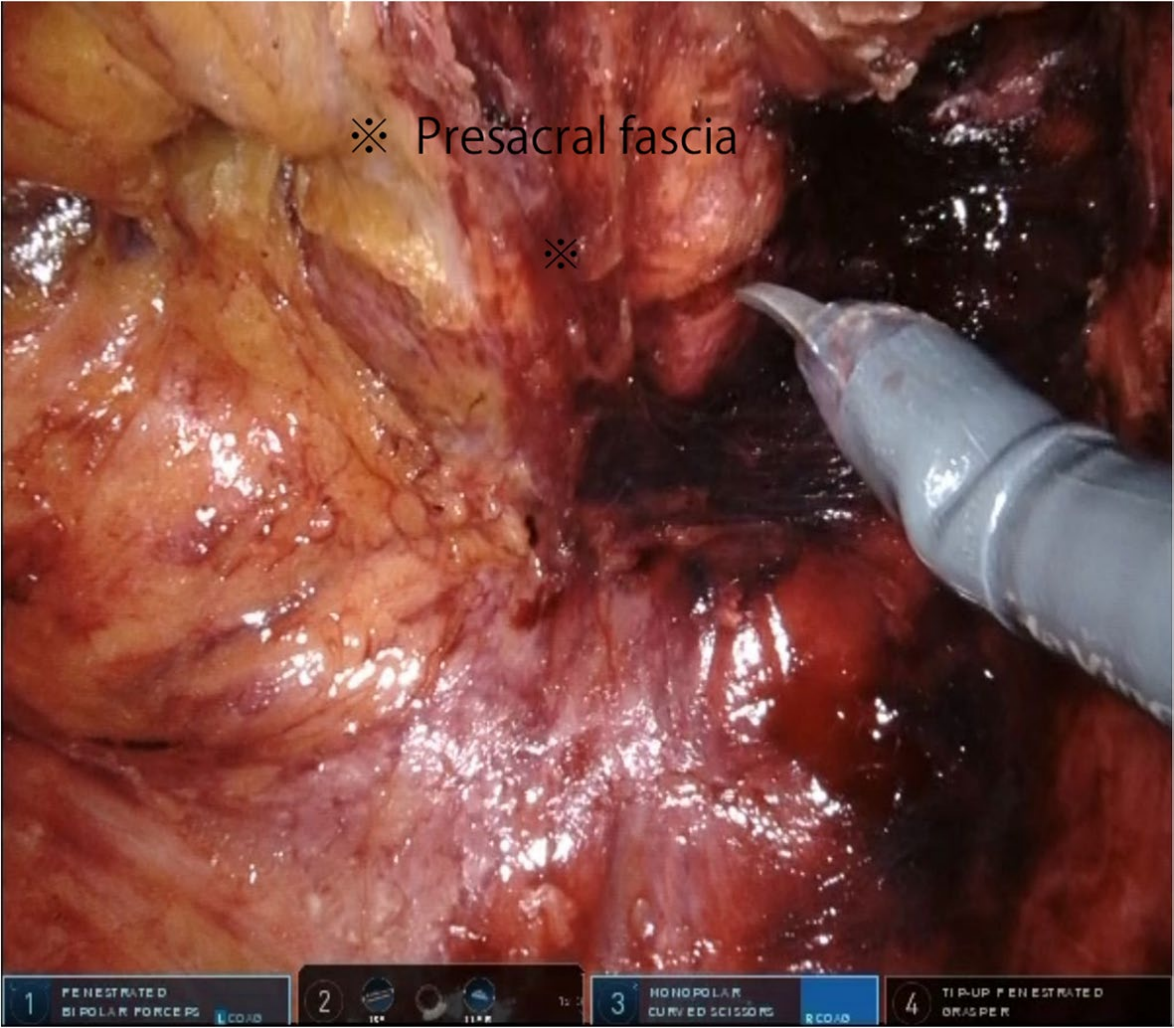
Visualizing gauze through elevation of the presacral fascia during an intra-abdominal surgical procedure

**Fig. 5:**
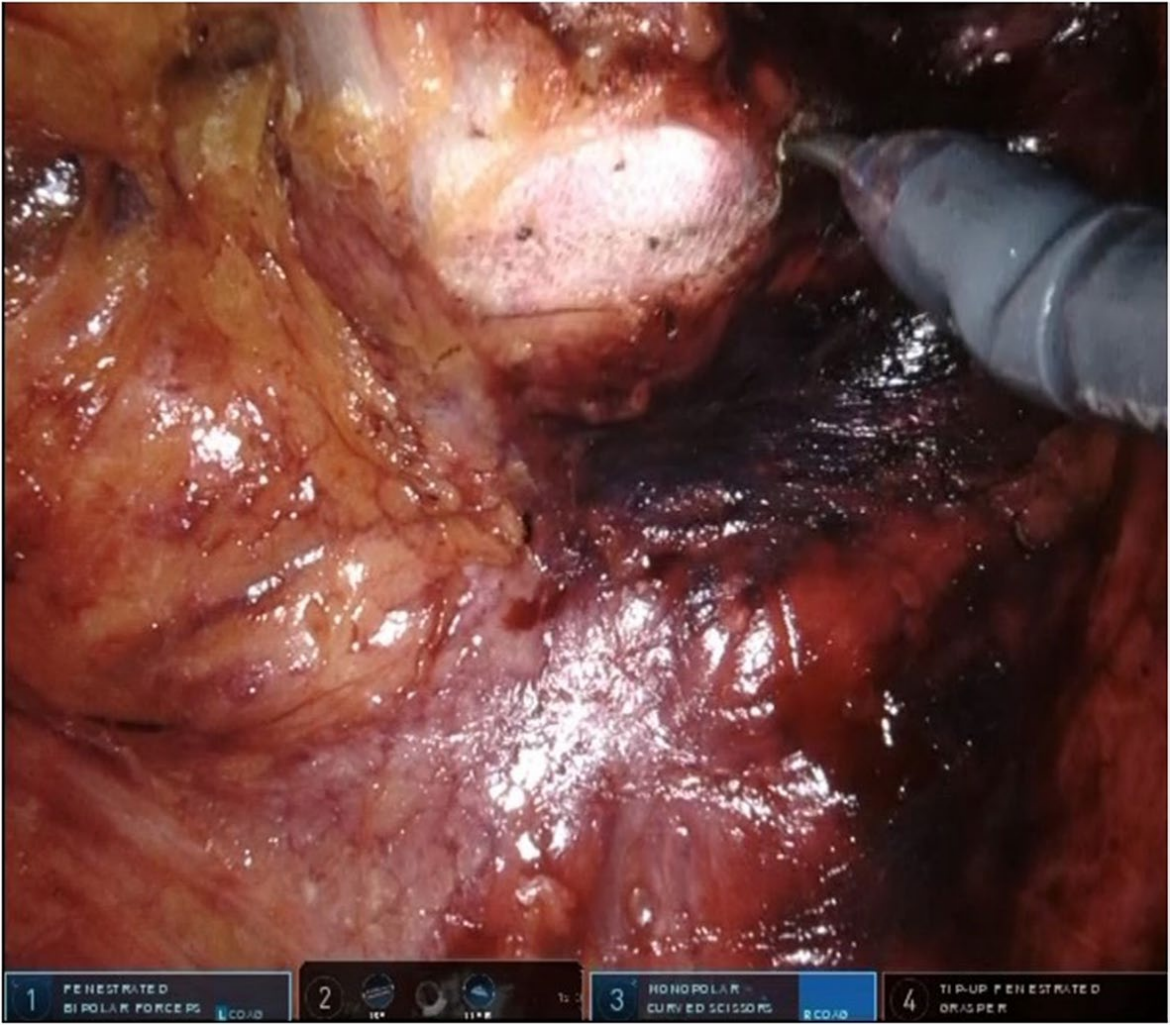
Incision of the presacral fascia during an intra-abdominal surgical procedure

1. The dorsal peritoneum of the inferior mesenteric artery was separated, and both the artery and vein were ligated and subsequently detached.
2. The descending colon and sigmoid colon were mobilized.
3. The rectum was mobilized using the total mesorectal excision method while a layer of visceral fascia was maintained, and the autonomic nervous system was preserved.
4. Mobilization of the rectum anteriorly was terminated below the level of the prostate (in males) or cervix (in women).
5. As the posterior mobilization of the rectum progressed, once it reached just anterior to the levator muscles, the gauze, which was placed at the conclusion of the first half of the perineal manipulation, became recognizable as the elevation of the presacral fascia [16] (Fig. 4).
6. The presacral fascia was sharply incised to create an opening toward the perineal region. Then, the colon was transected at least 10 cm from the rectal tumor on the oral side (Fig. 5).

### The latter half of perineal manipulation

1. The anterior dissection was performed by flipping the colonic stump to the perineal side for better visibility, and the specimen was removed.
2. The closure of the perineal incisions was made.

As these steps were completed, the assistants simultaneously finalized the abdominal closure and colostomy creation, bringing the entire surgery to a close.

## Results

Of the 99 patients who underwent robot-assisted rectal surgery at our hospital during the study period, nine were included in this study. The average age of the patients was 63.5 years. Of the nine patients, five were males. Table 1 shows the relevant patient characteristics. Patients 2, 6, and 8 received chemotherapy or radiation therapy before APR. Additionally, patient 2 underwent ileostomy construction before the chemotherapy. For this patient, the ileostomy was closed first, followed by the docking procedure.

**Table 1:**
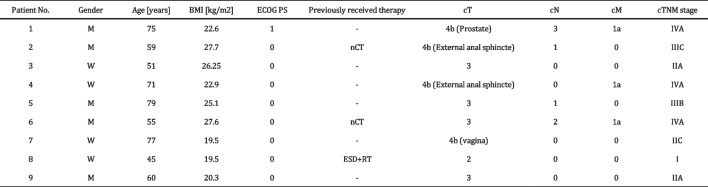
Patient demographics and clinical characteristics

*cT* Clinical T stage, *cN* Clinical N stage, *cM* Clinical M stage, *cTNM* Clinical TNM stage, *nCT* Neoadjuvant Chemoradiotherapy, *ESD* + *RT* Endoscopic Submucosal Dissection + Radiotherapy

Table 2 shows the operative and pathological findings. No injuries to other organs were observed, and no patients required conversion to open surgery. The median operation time was 231 (153–341) min, and the intraoperative blood loss was 170 (70–346) ml. Considering the last two cases where a trainee did not participate, the average operation time was 167.5 min with a blood loss of 80.5 ml. The median time of the first half of the perineal manipulation was 10 (6–15) min. Patient 8, who underwent combined resection of the vaginal wall, showed positive CRM. The median postoperative hospital stay was 16 (12–26) days. Surgical site infections, classified as Clavien–Dindo grade II complications, were observed in two cases: one at the drain removal site and the other at the pelvis. No patient experienced obvious urinary or erectile dysfunction. There were no cases of local recurrence or mortality observed during the study period, and the median local recurrence-free survival (LRFS) was 6.7 (1.2–15.0) months. One patient was undergoing chemotherapy for synchronous liver metastasis, and another patient underwent radical resection for metachronous liver metastasis.

**Table 2:**
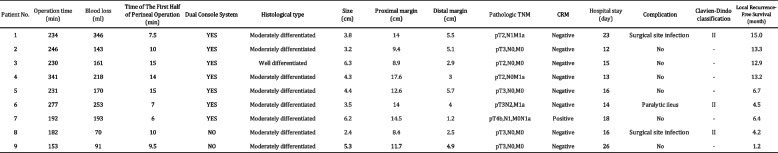
Operative and pathological characteristics

## Discussion

This study proposes an approach to enhance the efficiency of robot-assisted APR surgeries without an increase in personnel. This method has two main advantages. First, the surgeon can proactively perform part of the perineal manipulation while the bedside assistants are setting up the port, which optimizes the use of surgical time. Second, the dissection of the anococcygeal ligament during the aforementioned process clarifies the dissection point on the presacral fascia during console manipulation, facilitating a safer and easier incision. In this study, all our robot-assisted APR surgeries were successfully completed without conversion to open surgery or injury to other organs. Additionally, in the latter half of the perineal manipulation, our approach enables the bedside assistants to simultaneously finalize the abdominal closure and colostomy creation, bringing the entire surgery to a close. This contributes to time savings on abdominal closure and stoma construction, further optimizing the surgical procedure and enhancing overall efficiency. Moreover, our data indicated a median operation time of 231 min, which is reduced compared to certain previous studies on robot-assisted APR [2, 17]. Notably, in the last two cases where a trainee did not participate, the operation time further decreased to 167.5 min on average. Intraoperative blood loss was also within an acceptable range and was even lower (80.5 ml) in the last two cases. Previous studies have shown that surgical proficiency can affect intraoperative blood loss, which in turn influences postoperative morbidity and recovery [18, 19]. These facts suggest that our optimized approach has made the technically demanding procedure safer and less complicated.

Notably, applying the synchronous approach significantly reduces the need for a large surgical team. By optimizing the operation sequence and synchronizing the abdominal and perineal dissections, the involvement of excessive surgical staff could be minimized without compromising surgical efficiency or patient outcomes. Consequently, two robot-assisted surgeries can be conducted in a day using a single robot. This optimization of surgical resources contributes to the overall efficiency of the surgical department and further enhances the cost-effectiveness of robot-assisted APR.

Furthermore, this study suggests that starting with the resection of the anococcygeal ligament from the perineal side is a safe and versatile technique. It can be effectively applied not only in robotic surgeries but also in traditional open surgeries and laparoscopic procedures. By directly palpating the coccyx and creating an endpoint on the posterior aspect of the rectum during this stage, it is possible to avoid the holy plane [20], where the rectal mesentery tapers distally during posterior mobilization of the rectum. This allows for a proper connection with the perineal side without compromising the surgical margins.

Our efficient robot-assisted APR approach is the product of a collaboration between one operating surgeon and two bedside assistants. Their combined training efforts significantly contribute to reduced surgical times. It is important to stress the critical role of the training and development of bedside assistants in achieving these efficiency improvements. Not all bedside assistants can participate in this type of surgery without undergoing training beforehand. In our facility, the surgeon remains outside the sterile field during robot-assisted surgery, delegating all sterile operations to the bedside assistants. Our previous reports showed that the docking operation, unique to robot-assisted surgery, can be mastered after approximately 10 cases [21], making it accessible even to trainee doctors. Thus, our bedside assistants can acquire basic skills such as port placement, docking manipulation, adhesiolysis, and anvil head attachment. They have become proficient in resolving issues, such as port detachment, inadequate pneumoperitoneum, and re-docking, allowing the console surgeon to focus solely on the surgical procedure. Our efficient robot-assisted APR approach has achieved reproducible surgical outcomes due to these modifications.

This study showed a noticeable trend of prolonged hospitalization without medical necessity. We could not prove that our management techniques effectively reduced hospital stays. In Japan, the insurance system, including national health insurance and private insurance, significantly alleviates patients’ financial burdens compared to those in Western countries [22]. Therefore, patients in Japan often prioritize their families’ comfort and continuity of care within the hospital environment over reducing the length of their stay. This cultural preference presents challenges in meeting the targeted lengths of hospital stays, underscoring this as a key area for attention and improvement.

### Limitations

This study has some limitations. This was a single-center retrospective study, and a small number of patients were evaluated. Data were obtained from a single institution, potentially limiting the generalizability of our findings. Additionally, long-term oncologic outcomes were not analyzed in this study. Furthermore, this management approach carries specific limitations that must be considered. First, performing the approach is not recommended when anastomosis is possible. Second, this approach is not applicable when considering coccyx resection. Finally, the available evidence is inadequate to determine the appropriate skill level of bedside assistants who can participate in this approach. These limitations highlight the need for further investigation and careful consideration to optimize the application. Furthermore, these limitations indicate the necessity for further investigation, including studies across multiple institutions.

## Conclusion

The study results indicate that the challenges associated with APR can be efficiently addressed without requiring additional personnel by streamlining team composition and the synchronous approach. This optimization strategy minimizes the need for a larger surgical team, while maximizing the utilization of surgical time and resources.

## Data Availability

The datasets generated during and/or analyzed during the current study are available from the corresponding author on reasonable request.
